# Effect of proanthocyanidin mediated immediate and delayed dentin sealing on the strength of premolars restored with composite resin inlay

**DOI:** 10.4317/jced.55942

**Published:** 2020-03-01

**Authors:** Fereshteh Shafiei, Tayebeh Aghaei, Zahra Jowkar

**Affiliations:** 1 Professor, Oral and Dental Disease Research Center, Department of Operative Dentistry, School of Dentistry, Shiraz University of Medical Sciences, Shiraz, Iran; 2 Post-graduate Dental Student, Oral and Dental Disease Research Center, Department of Operative Dentistry, School of Dentistry, Shiraz University of Medical Sciences, Shiraz, Iran; 3 Assistant Professor, Oral and Dental Disease Research Center, Department of Operative Dentistry, School of Dentistry, Shiraz University of Medical Sciences, Shiraz, Iran

## Abstract

**Background:**

Immediate dentin sealing (IDS) with proanthocyanidin (PA) could be used before cementation with a self-adhesive (SA) cement. The aim of this study was to assess the effect of PA treatment on acid-etched dentin before adhesive application, in IDS and delayed dentin sealing (DDS), on the strengthening property of SA-cemented composite resin inlay in premolars.

**Material and Methods:**

Eighty-four maxillary premolars were divided into 7 groups (n=12): 1) (Intact) Sound teeth served as controls; groups 2-7) After cavity preparation and fabrication of composite resin inlay, temporary inlays were made and cemented. After one week, the inlays were removed and composite inlays were luted with a self-adhesive resin cement as follows: 2) (SA) Without dentin- pretreatment; 3) (DDS) Etch-and-rinse adhesive before the cementation; 4) (DDS/PA) PA treatment of acid-etched dentin before the adhesive, followed by the cementation; 5) (Etch/PA) PA treatment of acid-etched dentin before the cementation; 6 and 7) (IDS and IDS/PA) Application of IDS without or with PA treatment, respectively, one week before the cementation. After thermo-mechanical aging, fracture resistance (FR) was tested. Data were analyzed using one-way ANOVA and Tamhane tests (α=0.05).

**Results:**

There was a significant difference between the study groups (*P*<0.001). The IDS and IDS/PA groups yielded significantly higher FR compared to the SA group (*P* ≤ 0.003), but the DDS, DDS/PA and Etch/PA groups did not differ from the SA group (*P*>0.05). The effect of PA on FR in the IDS and DDS techniques was not significant.

**Conclusions:**

IDS with or without PA treatment considerably improved the strength of premolars with self-adhesive-cemented inlay, while the value of only IDS with PA treatment reached the level of the sound teeth.

** Key words:**Delayed dentin sealing, fracture resistance, immediate dentin sealing, proanthocyanidin.

## Introduction

An effective and stable bonding is mandatory between the resin cement and tooth structure in order to benefit from the strengthening effect of indirect adhesive restorations in teeth with severely weakened structure (1). Self-adhesive (SA) resin cements appear to be a proper choice to overcome the highly complex, technique sensitive and time-consuming nature of etch-and-rinse adhesive cementation (2,3). However, this simplification might be associated with lower bonding ability and the consequent strengthening effect (4,5). This is due to limited etching potential and superficial interaction of the highly viscous cement with tooth structure (3,4). SA resin cements contain acidic monomers that demineralize and infiltrate the tooth structure, creating micromechanical retention. Also, these monomers are capable of providing chemical bonding to hydroxyapatite (2,4). Despite this superficial interaction, their dentin bond strength was reported to be comparable to those of etch-and-rinse adhesive cements; however, enamel bonding ability was lower (3). On the other hand, freshly prepared and clean dentin, as an optimal substrate for bonding, is not available in the majority of indirect restorations. Following the provisional stage, dentin contaminated with residual temporary cement is not a suiTable substrate for bonding. The cement remnant is not easily removed with pumice/cleaning paste before final cementation (6). This is especially true for SA cement that does not need any pretreatment, compromising penetration of resin and bonding performance of SA cement (7). To overcome this, immediate dentin sealing (IDS) was suggested for etch-and-rinse adhesive resin cements in the early 1990s (8). The immediate application of a three-step or two-step etch-and-rinse system on freshly cut dentin before the impression making has demonstrated advantages in terms of bond strength, marginal adaptation/sealing, and post-cementation sensitivity (9,10). Apparently, this technique allows the dentin bond to develop without stresses induced by resin cement/restoration shrinkage (10). Oliveira *et al.* used a self-etching adhesive (Clearfil SE) for IDS and a SA resin cement (Panavia F) for the inlays cementation (11). They found that IDS had no effect on initial fracture resistance of premolars restored with composite resin inlay luted with Panavia F (11). On the other hand, Gresnigt *et al.* used Optibond FL (an etch & rinse adhesive) for the IDS and Variolink Veneer (a conventional resin cement) for the veneers cementation (12). They found that the fracture strength of laminate veneer was reported to increase following improved adhesion to large dentin exposure areas through IDS technique (12). This technique could protect cut dentin and pulp against bacterial leakage during the provisional phase (9). Moreover, the detachment of temporary restoration, saliva contamination, and microleakage during the temporary phase is highly possible. Therefore, including an antibacterial agent in the IDS technique could have beneficial effects. In addition to its antibacterial effect, the multifunctionality of proanthocyanidin from grape seed extract (PA) as a matrix metalloproteinase (MMP) inhibitor, collagen cross-linker, and antioxidant could make it attractive during cementation (13-15). The proanthocyanidin from grape seed extract (PA) is a flavanol compound that has a high affinity for proline-rich proteins, such as collagen. The interaction mechanism of PA with collagen is covalent and hydrophobic bonds, ionic interaction and hydrogen bonding. In additon to its antibacterial effect, multifunctionality of PA as a matrix metalloproteinase (MMP) inhibitor, collagen cross-linker and antioxidant, could make it attractive during cementation (13,15). PA can improve the elastic modulus and stiffness of the collagen matrix and increase the mechanical properties of demineralized dentin (13,15). In this sense, dentin pretreatment with PA was found to enhance the bond strength of etch-and-rinse (E&R) adhesives (15).

On the other hand, some authors suggested the application of E&R adhesive system before to SA cements to improve dentin bond strength (16). Therefore, PA in association with E&R adhesives could be applied just before to cementation with SA cement as delayed dentin sealing (DDS) or in the IDS method. However, the effects of the application of this modified IDS/DDS method in the efficacy of SA cements have not been evaluated in the literature. Therefore, this study was designed to examine the null hypothesis stating that IDS and DDS techniques combined with PA would have no effect on FR of premolars with an SA-cemented composite resin inlay after thermo-mechanical aging.

## Material and Methods

Following approval of the research protocol by the Research Ethics Committee of Shiraz University of Medical Sciences, 84 maxillary single-rooted premolars, extracted for orthodontic reasons, were selected. The teeth were sound with no defects, fractures or crack lines as verified under ×20 magnification. The samples were cleaned and disinfected in 0.5% chloramine solution and then stored in distilled water at 4ºC. The buccopalatal and mesiodistal dimensions of the teeth, measured with a digital caliper (Mitutoyo Digimatic; Mitutoyo, Kawasaki, Japan), were 9 and 7 mm, respectively, with a variation of 0.5 mm for each dimension. Before to embedding the teeth in a cylinder of self-curing acrylic resin up to 1 mm below the cementoenamel junction (CEJ), their roots were covered with a 0.2–0.3-mm layer of melted wax. This layer was replaced with a polyether impression material to mimic the periodontal ligament (17). The long axis of the tooth was perpendicular to the base of the cylinder. The teeth were randomly assigned to seven groups (n=12). The teeth included in group 1 remained intact, serving as a control group. A silicone mold was made from the premolar surface before to inlay preparation.

-MOD Inlay Preparation 

Standardized MOD cavities were prepared with conical round-ended diamond burs (#856-018-FG, Meisinger, USA) in a high-speed handpiece under water and air cooling. The preparations had rounded internal angles, 6° divergent walls, and an occlusal box with a width of two-thirds of the intercuspal distance and a buccopalatal dimension of 3.5±0.2 mm. The remaining buccal and palatal cusps had a thickness of 2.7±0.2 mm. The cervical wall was placed 1 mm above the CEJ in enamel, with a depth of 4±0.2 mm at the isthmus. The preparations had only buccal and palatal walls, with no axial walls. The diamond bur was replaced after every five preparations.

-Inlay Fabrication Temporary Phase and Cementation Procedures

Following isolation of the cavity surfaces with a medium of water-soluble gel (Johnson & Johnson, New Brunswick, NJ, USA), the composite inlays were fabricated with Z250 (3M ESPE, St. Paul, MN, USA) in four oblique incremental layers. Each layer was light-cured for 20 seconds with a halogen light-curing unit (Coltolux, Coltene Whaledent, Attstatten, Switzerland) at a light intensity of 500 mW/cm2. Light intensity output was checked every five restorations with a radiometer (Coltolux ® , Coltène/Whaledent Inc.,) from the same manufacturer. The composite inlays were then removed from the cavity and further polymerized in an oven (DI-500 Oven, Colten Whaledent) at 100ºC for 5 minutes. After air-particle abrasion of the internal surfaces of inlays with 50-µm alumina particles for 10 seconds, cleaning with ultrasonic and air-drying, a silane agent (Cimara Silane Coupling agent, Voco, Cuxhaven, Germany) and then a layer of the adhesive (Table 1) were applied.

**Table 1:**
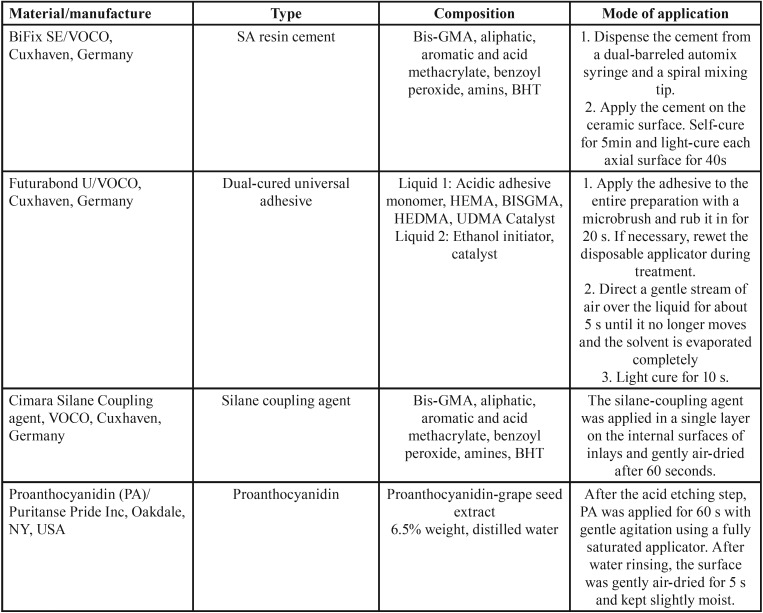
Materials used in this study.

Temporary inlays were fabricated using self-cured acrylic resin (Tempron, GC, Tokyo, Japan) and the silicone molds. The cavity surfaces were rinsed to remove the water-soluble gel that had been re-applied before temporary inlay fabrication.

In the groups 2-5, the inlay was cemented into the cavity immediately after preparation, using a non-eugenol temporary cement (Temp Bond NE, Kerr, USA) with a seating pressure for 2 minutes. In groups 6 and 7 (IDS groups), temporary cementation was performed in the same manner as described above, but after dentin sealing. Then, the specimens were stored in distilled water at 37ºC for one week. After removing the restoration from the cavity with a plastic instrument, excess cement was completely removed with a sickle explore. The surfaces were cleaned with pumice paste and a rotary rubber cup for 10 seconds.

A solution of 6.5% w/v PA was prepared by dissolving 6.5 g of PA-rich grape seed extract powder (Puritanse Pride Inc, Oakdale, NY, USA) in 100 mL of distilled water. In groups 4, 5 and 7, the prepared solution was applied on dentin surfaces for 60 seconds with gentle agitation, using fully saturated applicators.

Group 2 (SA): The mixed self-adhesive cement, Bifix SE (Voco) was applied to the fitting surfaces of the tooth and inlay using a self-mixing tip, and the inlay was cemented into the cavity without pretreatment.

Groups 3 (DDS): After acid-etching of the cavity surfaces with phosphoric acid for 15 seconds, rinsing and gentle air-drying, a dual-cured universal adhesive, Futurabond U (Voco) was applied for 20 seconds, air-dried and light-cured for 10 seconds. Then, cementation was conducted using Bifix SE.

Group 4 (DDS/PA): After acid-etching of the cavity surfaces with phosphoric acid for 15 seconds, rinsing and gentle air-drying, dentin surfaces were treated with the PA solution and rinsed with distilled water. Then, a dual-cured universal adhesive, Futurabond U (Voco), was applied for 20 seconds, air-dried and light-cured for 10 seconds. Then, cementation was conducted using Bifix SE.

Group 5 (Etch/PA): After acid-etching of the cavity surfaces, rinsing and gentle air-drying, PA solution was applied on the etched dentin. Then, cementation was performed using Bifix SE without the adhesive application.

Group 6 (IDS): Immediately after preparation and before temporization, the dentin was acid-etched with 37% phosphoric acid for 15 seconds, rinsed, air-dried, and sealed with Futurabond U (Table 1) in two layers. After one week and removal of temporary inlay/cement and cleaning, the enamel and sealed surfaces were acid-etched for 15 and 10 seconds, respectively and the inlay was cemented using Bifix SE.

Group 7 (IDS/PA): Immediately after preparation and before temporization, the dentin was acid-etched with 37% phosphoric acid for 15 seconds, rinsed, air-dried, and treated with the PA solution and sealed with Futurabond U in two layers. After one week and removal of temporary inlay/cement and cleaning, the enamel and sealed surfaces were acid-etched for 15 and 10 seconds, respectively and the inlay was cemented using Bifix SE.

In all the groups, the inlays were cemented under 1-Kg seating load for 5 minutes; after removing the excess cement with a microbrush, the light-curing procedure was performed using Coltulux light-curing unit (Coltene Whaledent) at 500 Mw/cm2 light intensity for 60 seconds from each side of the cemented teeth. All the cemented inlays were finished, polished and stored in distilled water at 37ºC for one week. The characteristics of the materials used in this study and their application procedures are presented in Table 1.

-Aging Procedures and Fracture Resistance Test

All the specimens were subjected to 100000 cycles of application of 50-N loading forces at a frequency of 0.5 Hz in a mastication simulation machine (Chewing Stimulator CS4; SD Mechatronic, Feldkirchen, Westerham, Germany) (18). The mechanical load was applied to the center of the occlusal surface in contact with both cusp ridges using a stainless steel antagonist with a rounded end that was 6 mm in diameter in a water environment. After thermal cycling for 1500 cycles at 5ºC/55ºC, the specimens were subjected to a compressive load at a crosshead speed of 1 mm/min in a universal testing machine (Zwick Roell, Ulm, Germany). The compressive load was applied parallel to the long axis of the tooth with a 6-mm-diameter stainless steel antagonist placed at the center of the tooth with contacts only on the buccal and palatal cuspal inclines. The peak force required for fracture was recorded in Newton as fracture strength (FR) value.

After confirmation of normal distribution of the data using the Kolmogorov-Smirnov test, they were analyzed with one-way ANOVA and Tamhane tests (α=0.05). Statistical analyses were performed using SPSS software version 17 (SPSS Inc, Chicago, IL, USA).

-Fracture Mode Evaluation

After FR testing, the specimens were assessed to classify the fracture modes as follows:

Mode I: cusp fracture extending to the CEJ/ Mode II: cusp fracture extending below the CEJ 

Mode III: restoration fracture along with cusp fracture at the CEJ/ Mode IV: restoration fracture along with cusp fracture extending below the CEJ / Mode V: longitudinal fracture dividing the tooth along the axis.

## Results

Table 2 presents the fracture resistance values in newton (mean ± SD) for the seven groups.

**Table 2:**
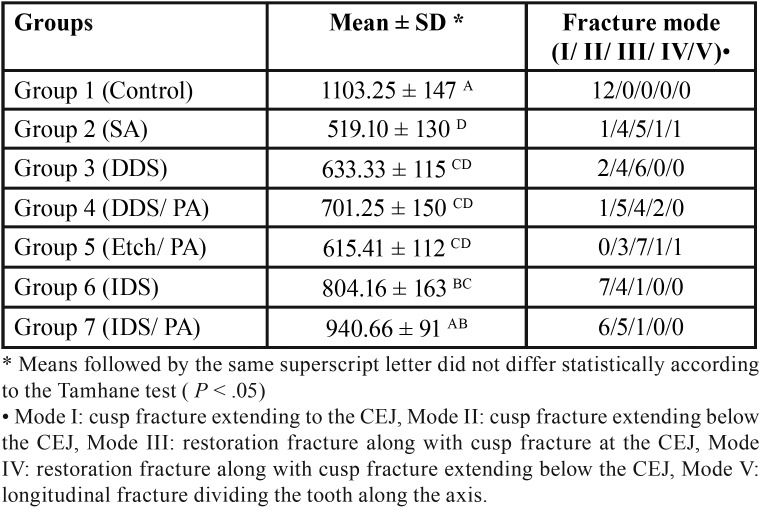
Fracture resistance in Newton (Mean ± SD) and fracture mode of the study groups.

According to one-way ANOVA, there were significant differences between the study groups (*P* < 0.001). Among the experimental groups, only FR in the IDS/PA group reached the level of the intact group. The two IDS groups had comparable FR values. The other groups exhibited significantly lower FR values compared to the IDS/PA and control groups (*P* ≤ 0.01, *P* ≤ 0.001, respectively). The lowest FR was obtained in the SA group, with no significant difference from the two DDS groups and etch/PA group (*P* > 0.05); however, it was significantly lower than those of the two IDS groups (*P* ≤ 0.003).

In the control group, mode I and in the two IDS groups, mode I and mode II fracture patterns were the predominant modes. In the other groups, most fractures were mode II and III (Table 2).

## Discussion

The present study assessed the performance of cementation with SA cement in terms of strengthening of weakened premolars following PA-mediated IDS and DDS techniques. When compared with the SA group (group 2), only the two IDS groups (groups 6 and 7) revealed a higher value of FR. Therefore, the null hypothesis could be partially rejected.

Previous studies on the efficacy of both IDS approaches and PA pretreatment were all dentin bond strength assessments (9,19-23). These tests were performed on flat small-surface areas of tooth structure; therefore, the effects of more complex inlay cavity and related C-factor and compliance of cavity design were not involved. Moreover, seating pressure and bur-prepared dentin surface of the cavity with a thicker and more compact smear layer *in vivo*, compared to 600-grit silicon carbide abraded dentin cannot mimic *in vitro* conditions. FR tests can include these factors in a better way (24). FR test used in this study is thought to provide a favorable simulation of chewing cycles on restored teeth and the clinical conditions. Simulation of chewing stresses was found to increase collagen degradation; collagen crosslinking agents might prevent this degradation (25). Therefore, the use of an MMP inhibitor agent might be beneficial for preserving bonding stability (26). In this study, PA-IDS (group 7) and then IDS (group 6) revealed the highest FR values. These values were significantly higher than those of corresponding DDS groups (4 and 3) and the SA group (group 2). Although FR of DDS groups, especially with PA, was higher than that of the SA group, the positive effect of DDS techniques was not statistically significant. These findings could confirm the important role of IDS technique, irrespective of PA pretreatment in achieving a higher FR.

Recently, the higher bond strength of SA cement was reported following IDS and DDS techniques with a universal adhesive in self-etch (SE) mode, although IDS was more significantly effective (21). Resin sealing of dentin with two-step SE increased bond strength of a self-adhesive cement (19). However, Ferreira-Filho *et al.* demonstrated higher bonding performance of IDS technique with one-step SE and three-step E&R compared to SA cement alone in the short term, but their bond strengths were the same after water aging (27).

Futurabond U used in this study has acidic pH, but dual-cured activator containing this two-component adhesive could prevent incompatibility between the cement and the adhesive in deep parts of the cavity in which the cement would cure through self-curing reaction (28). This universal adhesive was used in E&R mode, not SE mode. It is well known that the acid etching improves the enamel bonding, but it adversely affects the dentin bond strength (2-4). However, a previous study (Brigagão *et al.*) indicated that in the case of DDS technique, rinsing after etching (with acid or polyacrylic) could remove contaminants from the temporary cement and improve adhesion and bond strength to dentin (21). Moreover, since the bio-modification effect of PA is on demineralized dentin, it might not be applicable with SE adhesives (15). Accordingly, a PA/acid-etch group (with total etching of the enamel and dentin surfaces) was included in this study, based on the possible beneficial effect of acid-etching. Given that the exposed collagen matrix was reported to be prone to collapse due to cementation pressure (3,4), a collagen crosslinking agent (PA) was also tested in this study. Nevertheless, this approach, by itself, led to no beneficial effect in terms of FR. The high viscosity of the cement might limit its penetration into demineralized dentin, consequently this demineralized-non infiltrated collagen layer is prone to degradation (4). On the other hand, the acidic pH of SA cement could not be buffered by etched dentin, decreasing the degree of conversion of the cement (21). These factors might limit any positive effect of acid-etching on the removal of the smear layer and improvement of bond strength and collagen crosslinking of the exposed fibrils. In the present study, the IDS groups (6 and 7) yielded higher FR compared to that of the SA group (group 2). Although no significant difference was observed between the two IDS groups (with and without PA), only the FR of PA/IDS group was comparable with those of the intact group. This finding underlines the effectiveness of the IDS technique, while the application of PA has been beneficial only in combination with IDS.

Several studies have documented an improvements in the mechanical properties of PA-treated demineralized dentin (13-15). This was thought to result in improved bond strength of E&R adhesives (22,23). In addition to MMP inhibitory effect, PA leads to dehydration of collagen, i.e., the hydrophobic effect, consequently enhancing adhesive penetration and reduced water sorption (29,30). These could contribute to the stability of the hybrid layer and its resistance to MMPs’ activity (26). In the majority of these studies, the minimum application time of PA was 5 minutes (13-15,22,23). Some authors, by focusing on clinically applicable treatment time, reported a beneficial effect of PA-treatment for 60 and 120 seconds on crosslinking degree and tensile strength of demineralized dentin with the highest effect for 120 seconds at 15% concentration (29). In addition, this effect on resin–dentin bond strength was reported with 10% and 15% concentration (30). These improvements revealed a concentration- and time-dependent manner (29). Nevertheless, increased dentin bond strength stability and reduced collagen activity of MMPs was demonstrated with 60-second PA treatment (26). Even improved biological stability of the cross-linked collagen was confirmed in times as short as the 10-second PA application (31). In these reports, different application time (s)/concentration (s) of PA, the solvent of PA solution, adhesive composition, and experimental set-ups, such as bond strength, dentin mechanical properties and collagenase activity, were employed. No study has reported the effect of PA treatment on the strength of the cemented teeth. Differences in these contributing factors between our study and the reported studies might justify differences in the outcomes.

The present study was conducted on the extracted teeth, using a SA resin cement *in vitro*. Therefore, the results cannot directly be extended to the clinic and other SA cements with different compositions. Long-term *in vitro* studies and clinical trials are required to elucidate the effect of PA treatment during IDS on the performance of SA resin cements.

In light of the results of this FR study, generally PA treatment in two dentin sealing methods led to no significant effect; however, IDS, along with PA, resulted in considerable strengthening effect. Therefore, clinicians could benefit from reduced sensitivity during temporization and enamel acid-etching before final SA cementation without concern about inadvertent dentin etching.

## References

[1] Spitznagel FA, Horvath SD, Guess PC, Blatz MB (2014). Resin bond to indirect composite and new ceramic/polymer materials: a review of the literature. J Esthet Dent.

[2] Radovic I, Monticelli F, Goracci C, Vulicevic ZR, Ferrari M (2008). Self-adhesive resin cements: a literature review. J Adhes Dent.

[3] Hikita K, Van Meerbeek B, De Munck J, Ikeda T, Van Landuyt K, Maida T (2007). Bonding effectiveness of adhesive luting agents to enamel and dentin. Dent Mater.

[4] De Munck J, Vargas M, Van Landuyt K, Hikita K, Lambrechts P, Van Meerbeek B (2004). Bonding of an auto-adhesive luting material to enamel and dentin. Dent Mater.

[5] Monticelli F, Osorio R, Mazzitelli C, Ferrari M, Toledano M (2008). Limited decalcification/diffusion of self-adhesive cements into dentin. J Dent Res.

[6] Kanakuri K, Kawamoto Y, Matsumura H (2005). Influence of temporary cement remnant and surface cleaning method on bond strength to dentin of a composite luting system. J Oral Sci.

[7] Takimoto M, Ishii R, Iino M, Shimizu Y, Tsujimoto A, Takamizawa T (2012). Influence of temporary cement contamination on the surface free energy and dentine bond strength of self-adhesive cements. J Dent.

[8] Pashley EL, Comer RW, Simpson MD, Horner JA, Pashley DH, Caughman WF (1992). Dentin permeability: sealing the dentin in crown preparations. Oper Dent.

[9] Duarte Jr S, de Freitas CRB, Saad JRC, Sadan A (2009). The effect of immediate dentin sealing on the marginal adaptation and bond strengths of total-etch and self-etch adhesives. J Prosthet Dent.

[10] Qanungo A, Aras MA, Chitre V, Mysore A, Amin B, Daswani SR (2016). Immediate dentin sealing for indirect bonded restorations. J Prosthodont Res.

[11] Oliveira L, Mota E, Borges G, Burnett Jr L, Spohr A (2014). Influence of immediate dentin sealing techniques on cuspal deflection and fracture resistance of teeth restored with composite resin inlays. Oper Dent.

[12] Gresnigt MM, Cune MS, de Roos JG, Özcan M (2016). Effect of immediate and delayed dentin sealing on the fracture strength, failure type and Weilbull characteristics of lithiumdisilicate laminate veneers. Dent Mater.

[13] Epasinghe D, Yiu C, Burrow M, Tsoi J, Tay F (2014). Effect of flavonoids on the mechanical properties of demineralised dentine. J Dent.

[14] Bedran-Russo AK, Pauli GF, Chen S N, McAlpine J, Castellan CS, Phansalkar RS (2014). Dentin biomodification: strategies, renewable resources and clinical applications. Dent Mater.

[15] Balalaie A, Rezvani MB, BASIR MM (2018). Dual function of proanthocyanidins as both MMP inhibitor and crosslinker in dentin biomodification: A literature review. Dent Mater J.

[16] Barcellos DC, Batista GR, Silva M, Rangel PM, Torres C, Fava M (2011). Evaluation of bond strength of self-adhesive cements to dentin with or without application of adhesive systems. J Adhes Dent.

[17] Shafiei F, Doozandeh M, Ghaffaripour D (2019). Effect of Different Liners on Fracture Resistance of Premolars Restored with Conventional and Short Fiber-Reinforced Composite Resins. J Prosthodont.

[18] Kalay TS, Yildirim T, Ulker M (2016). Effects of different cusp coverage restorations on the fracture resistance of endodontically treated maxillary premolars. J Prosthet Dent.

[19] Sailer I, Oendra AEH, Stawarczyk B, Hämmerle CH (2012). The effects of desensitizing resin, resin sealing, and provisional cement on the bond strength of dentin luted with self-adhesive and conventional resincements. J Prosthet Dent.

[20] Hironaka NG, Ubaldini AL, Sato F, Giannini M, Terada RS, Pascotto RC (2018). Influence of immediate dentin sealing and interim cementation on the adhesion of indirect restorations with dual-polymerizing resin cement. J Prosthet Dent.

[21] Brigagão VC, Barreto LF, Gonçalves KA, Amaral M, Vitti RP, Neves AC (2017). Effect of interim cement application on bond strength between resin cements and dentin: Immediate and delayed dentin sealing. J Prosthet Dent.

[22] Srinivasulu S, Vidhya S, Sujatha M, Mahalaxmi S (2012). Shear bond strength of composite to deep dentin after treatment with two different collagen cross-linking agents at varying time intervals. Oper Dent.

[23] Al-Ammar A, Drummond JL, Bedran-Russo AK (2009). The use of collagen cross-linking agents to enhance dentin bond strength. J Biomed Mater Res B Appl Biomater.

[24] Saikaew P, Chowdhury AA, Fukuyama M, Kakuda S, Carvalho RM, Sano H (2016). The effect of dentine surface preparation and reduced application time of adhesive on bonding strength. J Dent.

[25] Turco G, Frassetto A, Fontanive L, Mazzoni A, Cadenaro M, Di Lenarda R (2016). Occlusal loading and cross-linking effects on dentin collagen degradation in physiological conditions. Dent Mater.

[26] Hass V, Luque-Martinez IV, Gutierrez MF, Moreira CG, Gotti VB, Feitosa VP (2016). Collagen cross-linkers on dentin bonding: stability of the adhesive interfaces, degree of conversion of the adhesive, cytotoxicity and in situ MMP inhibition. Dent Mater.

[27] Ferreira-Filho R, Ely C, Amaral R, Rodrigues J, Roulet J, Cassoni A (2018). Effect of Different Adhesive Systems Used for Immediate Dentin Sealing on Bond Strength of a Self-Adhesive Resin Cement to Dentin. Oper Dent.

[28] Alex G (2015). Universal adhesives: the next evolution in adhesive dentistry. Compend Contin Educ Dent.

[29] Liu R, Fang M, Xiao Y, Li F, Yu L, Zhao S (2011). The effect of transient proanthocyanidins preconditioning on the cross-linking and mechanical properties of demineralized dentin. J Mater Sci Mater Med.

[30] Liu Y, Chen M, Yao X, Xu C, Zhang Y, Wang Y (2013). Enhancement in dentin collagen's biological stability after proanthocyanidins treatment in clinically relevant time periods. Dent Mater.

[31] Fang M, Liu R, Xiao Y, Li F, Wang D, Hou R (2012). Biomodification to dentin by a natural crosslinker improved the resin-dentin bonds. J Dent.

